# The Association Between Hyperketonemia and Reduced Circulating Hydrogen Sulfide in Children with Type 1 Diabetes, and Ketone Inhibition of Cystathionine-γ-Lyase mRNA Expression in Cultured Hepatocytes

**DOI:** 10.3390/genes17091090

**Published:** 2026-09-10

**Authors:** Sushil K. Jain, Rajesh Parsanathan, Jeffrey Justin Margret, Neslihan Gungor, John A. Vanchiere, Maroun J. Mhanna, Robert McVie

**Affiliations:** 1 Department of Pediatrics, Louisiana State University Health Shreveport, Shreveport, LA 71103, USA; rajesh.uom@gmail.com (R.P.); jeffrey.justinmargret@lsuhs.edu (J.J.M.); neslihan.gungor@lsuhs.edu (N.G.); john.vanchiere@lsuhs.edu (J.A.V.); maroun.mhanna@lsuhs.edu (M.J.M.); robert.mcvie@lsuhs.edu (R.M.); 2 Department of Biotechnology, School of Integrative Biology, Central University of Tamil Nadu, Neelakudi, Thiruvarur 610005, India

**Keywords:** CSE, hydrogen sulfide, type 1 diabetes, vitamin D, HDL-cholesterol, hepatocytes, gene expression, hyperglycemia, hyperketonemia, ketone

## Abstract

Background: Hydrogen sulfide (H_2_S) is known for its significant anti-inflammatory, antioxidant, and vasculoprotective properties. In diabetic patients, blood levels of H_2_S are observed to be lower. Type 1 diabetic patients (T1D) often experience both hyperketonemia and hyperglycemia. Cystathionine-γ-lyase (CSE), an enzyme encoded by the *CTH* gene, is responsible for the biosynthesis of H_2_S. However, it remains uncertain whether the downregulation of *CTH* genes by ketones is linked to reduced levels of H_2_S. Objective: This study aims to test the hypothesis that ketones decrease *CTH* gene expression in cultured hepatocytes and that hyperketonemia negatively correlates with H_2_S levels in T1D. Methods: Fasting blood samples were collected from children with T1D (*n* = 36) and age-matched healthy controls (*n* = 35), following written informed consent. Plasma levels of H_2_S, 25(OH)VD, ketones, and inflammatory biomarkers were analyzed. To assess the effects of hyperglycemia and hyperketonemia on *CTH* gene expression, cultured hepatocytes were exposed to high glucose, acetoacetate, or β-hydroxybutyrate. Results: Blood levels of H_2_S and 25(OH)VD were significantly lower in T1D patients compared to healthy controls. Plasma H_2_S concentrations showed an inverse association with total ketones, BHB, acetoacetate, and MMP-9, while they were positively associated with HDL-cholesterol and 25(OH)VD. In cultured hepatocytes, exposure to acetoacetate and high glucose was linked to reduced *CTH* mRNA expression. Conclusions: Lower circulating H_2_S concentrations are associated with hyperketonemia and inflammatory/metabolic abnormalities in children and adolescents with T1D. The *in vitro* findings suggest that high glucose and acetoacetate may affect *CTH* transcription; however, further studies measuring CSE protein, enzymatic activity, and H_2_S production are necessary to clarify the underlying mechanism and establish causality.

## 1. Introduction

Type 1 diabetes (T1D) is an autoimmune disorder characterized by the destruction of pancreatic β-cells, leading to absolute insulin deficiency and persistent hyperglycemia. Unlike type 2 diabetes (T2D), T1D is also associated with hyperketonemia, which results from increased hepatic ketogenesis due to insulin deficiency [1]. In uncontrolled cases of T1D, circulating ketone body concentrations can reach approximately 25 mM [2]. While ketone bodies serve as important alternative energy sources during periods of glucose deficiency, their excessive accumulation can lead to various adverse biological effects, including increased oxidative stress, inflammation, and vascular dysfunction [3]. Cardiovascular complications pose a significant long-term concern for individuals with T1D, although the mechanisms linking metabolic dysregulation to vascular injury may differ from those observed in T2D [4,5].

Hydrogen sulfide (H_2_S) is an endogenous gasotransmitter that plays a crucial role in maintaining cardiovascular homeostasis due to its anti-inflammatory, antioxidant, and vasoprotective properties [6,7]. Reduced circulating levels of H_2_S have been observed in various conditions associated with vascular dysfunction and inflammation, such as hypertension and T2D [8,9]. Blood H_2_S levels are significantly lower in fasting samples from patients with type 2 diabetes compared to age-matched healthy subjects, as well as in streptozotocin-treated diabetic rats compared to control Sprague-Dawley rats [10]. Enzymatic H_2_S biosynthesis is impaired in a murine model of type 1 diabetes, and reduced H_2_S bioavailability is associated with impaired vascular reactivity [11]. Native thiol levels and the native thiol/total thiol ratio are significantly lower in T1D patients than in the control group [12]. Additionally, thiol/disulfide homeostasis is disrupted in the blood of pediatric diabetic patients [13,14,15].

Cystathionine γ-lyase (CSE), a key enzyme encoded by the *CTH* gene, is responsible for H_2_S production in the cardiovascular system and plays a significant role in vascular H_2_S biosynthesis [6]. Experimental studies indicate that deficiencies in *CTH* gene expression can lead to decreased endogenous H_2_S production and contribute to the formation of early atherosclerotic lesions, often accompanied by elevated plasma cholesterol and LDL-cholesterol levels [16]. Conversely, administering H_2_S donors has been shown to reduce vascular inflammation and impede the development of atherosclerotic lesions in experimental models [17,18]. Collectively, these findings underscore the critical role of impaired *CTH* gene expression and H_2_S signaling in vascular dysfunction and atherosclerosis.

The role of H_2_S in T1D, particularly in relation to hyperketonemia, remains insufficiently understood, despite its recognized cardiovascular protective effects. T1D is characterized by hyperglycemia and increased ketone production during insulin deficiency, with emerging evidence indicating that ketone bodies may influence cellular redox and inflammatory pathways [2]. Livers from streptozotocin-treated T1D rats exhibit significantly higher production of reactive oxygen species, alongside decreased CSE protein expression and activity, resulting in reduced H_2_S formation compared to controls. Studies involving T1D patients have shown a decrease in CSE protein expression and activity in peripheral blood mononuclear cells (PBMCs) compared to age-matched normal subjects [19]. However, the relationship between circulating ketone levels, H_2_S concentrations, and inflammatory biomarkers in children with T1D has yet to be thoroughly investigated. It remains unclear whether the downregulation of *CTH* gene expression by ketones correlates with reduced circulating H_2_S levels.

This study aims to explore the hypothesis that ketones downregulate *CTH* using cultured hepatocytes and that hyperketonemia negatively impacts circulating H_2_S levels in patients with T1D. This study has measured fasting circulating H_2_S levels in children with T1D and examined their relationship with hyperketonemia and cardiometabolic risk factors. Additionally, we have investigated how the primary ketone body, acetoacetate, influences *CTH* gene expression in cultured hepatocytes to explore a potential molecular mechanism through which hyperketonemia may reduce H_2_S bioavailability. Enhancing our understanding of the interplay between ketone metabolism and H_2_S signaling could shed light on the mechanisms underlying excessive vascular inflammation and the associated risk of vascular disease in T1D, potentially identifying new therapeutic targets to prevent diabetic vascular complications.

## 2. Materials and Methods

### 2.1. Study Participants

This case–control study enrolled 36 children and adolescents diagnosed with T1D and 35 healthy control subjects, all aged between 8 and 21 years. The study protocol received approval from the Institutional Review Board, with Protocol #H13-030 for diabetic children and Protocol #H13-029 for healthy children. Written informed consent was obtained from all participants or their parents/legal guardians before enrollment. T1D was confirmed through a clinical diagnosis supported by evidence of hyperglycemia, ketosis, low or absent C-peptide concentrations, insulin dependence, and the presence of pancreatic islet autoantibodies. Healthy controls were recruited from families visiting the pediatric diabetes clinic during the study recruitment period. Controls were screened to exclude diabetes and other chronic illnesses and were frequency-matched to the T1D group with respect to age and sex where applicable. Exclusion criteria included participants with renal disease, liver disease, sickle cell disease, pregnancy, or lactation. Additionally, subjects receiving vitamin supplements, antioxidant preparations, or herbal products were also excluded.

### 2.2. Blood Collection and Biochemical Measurements

Following an overnight fast of at least 8 h, venous blood samples were collected into serum and EDTA-containing tubes. Serum samples were submitted to the clinical laboratory for routine biochemical analyses, whereas EDTA plasma was separated by centrifugation at 1500× *g* for 15 min and stored at −80 °C until analysis. Fasting plasma glucose, HbA1c, lipid profile, liver enzymes, blood urea nitrogen, and creatinine were measured using standard clinical laboratory procedures.

### 2.3. Measurement of 25-Hydroxyvitamin D and MMP-9

Circulating concentrations of 25-hydroxyvitamin D [25(OH)VD] and matrix metalloproteinase-9 (MMP-9) were determined using commercially available sandwich ELISA kits (Thermo Fisher Scientific, Rockford, IL, USA) according to the manufacturer’s instructions. Standards and quality-control samples supplied with the kits were included in every assay, and all samples were analyzed in duplicate.

### 2.4. Measurement of Ketone Bodies

Plasma acetoacetate and β-hydroxybutyrate (BHB) concentrations were measured according to the methods described by Artuch et al. [20] and Koch and Feldbruegge [21]. Total ketone concentrations were calculated for each participant as the sum of acetoacetate and BHB after conversion to a common unit of mM. Type 1 diabetic patients with total ketone concentrations ≤25.0 mM were classified as normoketonemic (NK), whereas those with concentrations >25.0 mM were classified as hyperketonemic (HK). Group assignment was performed after completion of all biochemical analyses.

### 2.5. Determination of Hydrogen Sulfide

Plasma H_2_S concentrations were quantified using the methylene blue method outlined by Zhu et al. [22]. Frozen plasma aliquots stored at −80 °C and not previously thawed were used for the analysis. To minimize potential changes in sulfide concentrations during sample handling, samples were processed under consistent conditions and analyzed in duplicate. Plasma specimens were treated with zinc acetate to capture sulfide as zinc sulfide, subsequently reacting with N,N-dimethyl-p-phenylenediamine sulfate and ferric chloride in acidic conditions to produce methylene blue. Proteins were isolated through the application of trichloroacetic acid, followed by centrifugation of the samples at 10,000× *g* for a duration of 10 min. Absorbance was quantified at 670 nm utilizing a microplate reader. H_2_S levels were determined using a sodium sulfide standard curve ranging from 0 to 62.5 μM. The plasma H_2_S levels recorded in our investigation fell within the spectrum of circulating H_2_S concentrations documented in prior human studies by others [23,24].

Because the methylene blue assay involves chemical trapping of sulfide and measurement after sample processing, the resulting concentration represents an operational measure of the plasma sulfide pool captured under the assay conditions rather than a direct measurement of free dissolved H_2_S gas. Accordingly, associations involving circulating H_2_S are interpreted as associations with the measured plasma H_2_S pool rather than definitive evidence of changes in free bioactive H_2_S.

### 2.6. Cell Culture

The human hepatocyte cell line HepG2 (ATCC, Manassas, VA, USA) was cultured in RPMI-1640 medium supplemented with 10% heat-inactivated fetal bovine serum, 100 U/mL penicillin, 100 μg/mL streptomycin, 12 mM sodium bicarbonate, 12 mM HEPES, and 2 mM L-glutamine in a humidified incubator containing 5% CO_2_ at 37 °C. Before treatment, cells were washed with serum-free RPMI-1640 and cultured in complete medium.

### 2.7. High Glucose and Ketone Treatments

HepG2 cells were used to independently examine the effects of glucose, acetoacetate, and BHB on *CTH* mRNA expression. For the glucose experiment, cells were exposed to either normal glucose (7 mM) or high glucose (25 mM) for 24 h. For the AA dose–response experiment, cells were exposed to AA at 2, 4, or 6 mM for 24 h. For the BHB experiment, cells were exposed to BHB at 3, 6, or 12 mM for 24 h. Appropriate untreated/control cells were maintained under the corresponding basal culture conditions. These were conducted as separate treatment experiments. Each treatment condition was evaluated using three independent biological replicates (*n* = 3). Mannitol (18 mM) was used as an osmotic control for the high-glucose treatment. Cell viability following treatment was assessed using the Alamar Blue assay (Alamar Biosciences, Sacramento, CA, USA) according to the manufacturer’s protocol.

### 2.8. Quantitative Real-Time PCR

Following treatment, the cells were harvested, and total RNA was isolated using the RNeasy Mini Kit (Qiagen, Hilden, Germany). RNA concentration and purity were assessed using a NanoDrop spectrophotometer (Thermo Scientific, Swedesboro, NJ, USA). One microgram of total RNA was reverse transcribed using the High-Capacity RNA-to-cDNA Reverse Transcription Kit (Applied Biosystems, Waltham, MA, USA). Quantitative real-time PCR was performed using RT^2^ SYBR Green Master Mix (Qiagen) on an Opus 384 Real-Time PCR System (Bio-Rad, Hercules, CA, USA). Gene-specific primers were designed with Primer3-BLAST (version 2.5.0) and subsequently synthesized by Invitrogen, Carlsbad, CA, USA. The primer sequences used for *CTH* amplification are provided in Table 1. Each qPCR run included a no-template control and reverse-transcription negative control to monitor contamination and genomic DNA amplification. Amplification specificity was evaluated by melt-curve analysis. Relative gene expression was calculated using the 2^−ΔΔCt^ method and normalized to *GAPDH*.

All chemicals were purchased from Sigma Chemical Co. (St. Louis, MO, USA) unless otherwise mentioned.

### 2.9. Statistical Analysis

Every plasma sample was examined in duplicate, with fluctuations in the analysis of identical samples remaining below 6%. The plasma levels of various biochemical assessments fell within the concentration ranges of corresponding standards that were processed concurrently and supplied by the manufacturer’s kit. Statistical analyses were conducted using SigmaStat v4.0 (Systat Software Inc., San Jose, CA, USA). Continuous variables are expressed as mean ± standard error (SE). Prior to statistical testing, data distributions were assessed. Comparisons between two independent groups were made using an unpaired Student’s *t*-test for normally distributed variables or the Mann–Whitney U test for non-normally distributed variables. For comparisons among more than two groups, one-way analysis of variance (ANOVA) was utilised, followed by the Student–Newman–Keuls post hoc test for normally distributed data or the Kruskal–Wallis test for nonparametric data. Multivariable analyses were conducted to determine whether the principal associations persisted after accounting for potential confounding variables, including age, HbA1c, and duration of diabetes. The correlation coefficient (r) presented in the figures was derived from multiple linear regression analysis, incorporating HbA1c, diabetes duration, BMI, and the subjects’ age as additional variables. Given the relatively small sample size, particularly in the hyperketonemic subgroup, the adjusted analyses were interpreted as exploratory. A *p*-value of 0.05 or lower was considered statistically significant.

## 3. Results

The demographic and clinical characteristics of the study participants are summarized in Table 2. Healthy controls and patients with T1D were comparable with respect to age, sex, race, and body mass index (BMI). Fasting plasma glucose, HbA1c, acetoacetate, BHB, and total ketone concentrations were significantly higher in the T1D group than in healthy controls. Circulating H_2_S and 25(OH)VD concentrations were significantly lower in patients with T1D than in healthy controls.

The C-reactive protein (CRP), TNF-α, and H_2_S levels were statistically significant (*p* < 0.05) between the healthy control and T1D groups. Comparison of the normoketonemic and hyperketonemic groups demonstrated statistically significant differences (*p* < 0.05) in CRP, HDL-cholesterol, and H_2_S, whereas MMP-9, TNF-α, and triglycerides were not significantly different between the groups (Table 3).

**Figure 1:**
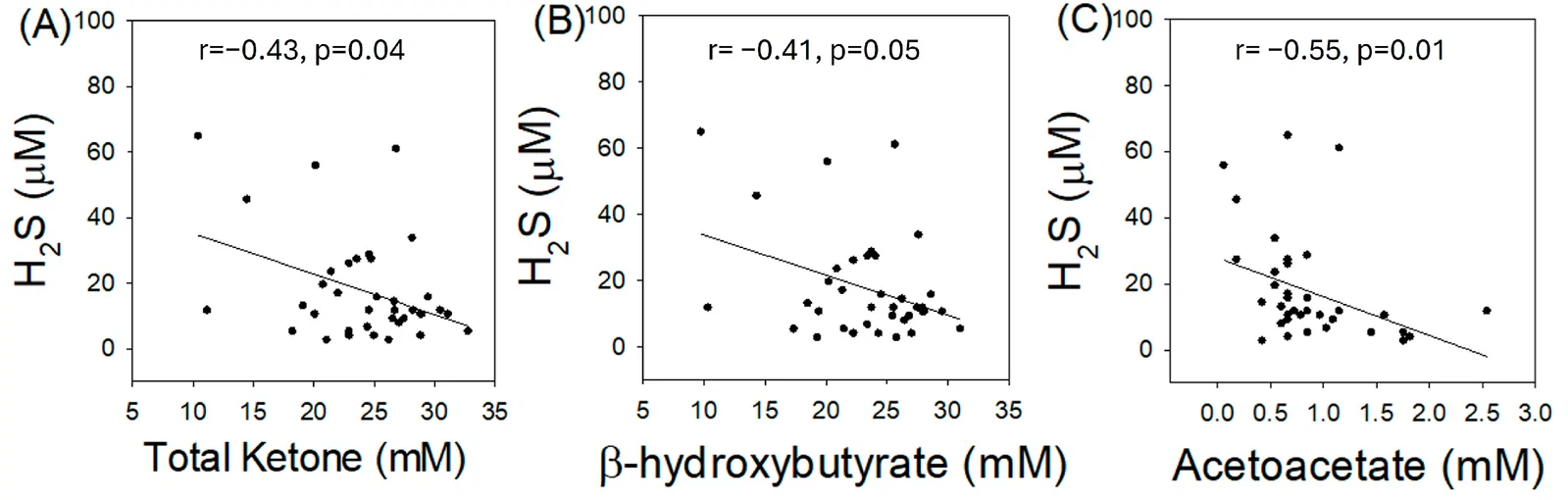
The plot diagram shows a negative association between plasma H_2_S and the levels of (**A**) total ketones, (**B**) β-hydroxybutyrate, and (**C**) acetoacetate. The correlation coefficient (r) given in the figures was obtained using multiple linear regression analysis conducted incorporating HbA1c, duration of diabetes, BMI, and age of subjects as additional variables.

Figure 1 shows a negative association between plasma H_2_S and the levels of (A) total ketones, (B) β-hydroxybutyrate, and (C) acetoacetate in patients with T1D. The correlation coefficient (r) given in the figures was obtained using multiple linear regression analysis conducted incorporating HbA1c, duration of diabetes, BMI, and age of subjects as additional variables. Plasma H_2_S concentrations were negatively correlated with acetoacetate (r = −0.55, *p* = 0.01), BHB (r = −0.41, *p* = 0.05), and total ketone concentrations (r = −0.43, *p* = 0.04). In contrast, circulating 25(OH)VD concentrations were not significantly associated with acetoacetate, BHB, or total ketone levels.

### 3.1. Associations Among H2S, 25(OH)VD, Inflammatory Markers, and Lipid Profile

**Figure 2:**
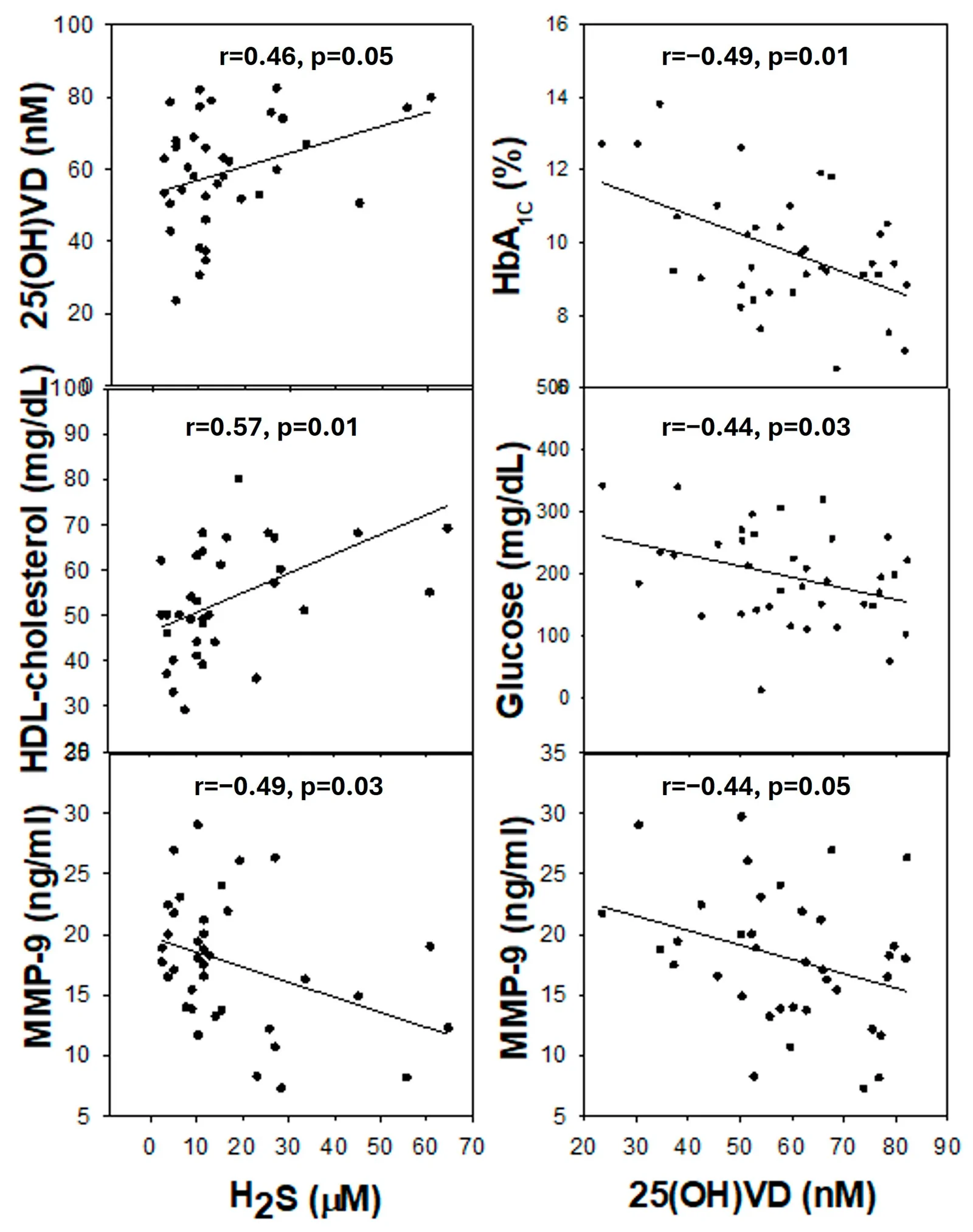
Plot diagram showing the association of plasma H_2_S levels with 25(OH)VD, HDL-cholesterol, and MMP-9, and the negative association of 25(OH)VD with HbA1C, glucose, and MMP-9 levels. The correlation coefficient (r) given in the figures was obtained using multiple linear regression analysis conducted incorporating HbA1C, duration of diabetes, and age of subjects as additional variables.

The associations among H_2_S, 25(OH)VD, inflammatory biomarkers, and lipid parameters are shown in Figure 2. Plasma H_2_S concentrations were positively correlated with 25(OH)VD (r = 0.46; *p* = 0.05) and HDL-cholesterol (r = 0.57; *p* = 0.01), whereas they were negatively correlated with MMP-9 (r = −0.49; *p* = 0.03). Likewise, circulating 25(OH)VD concentrations were inversely associated with HbA1c (r = −0.49; *p* = 0.01), fasting glucose (r = −0.44; *p* = 0.03), and MMP-9 (r = −0.44; *p* = 0.05) levels.

### 3.2. High Glucose, Acetoacetate, and BHB Are Associated with Reduced CTH mRNA Expression in HepG2 Cells

**Figure 3:**
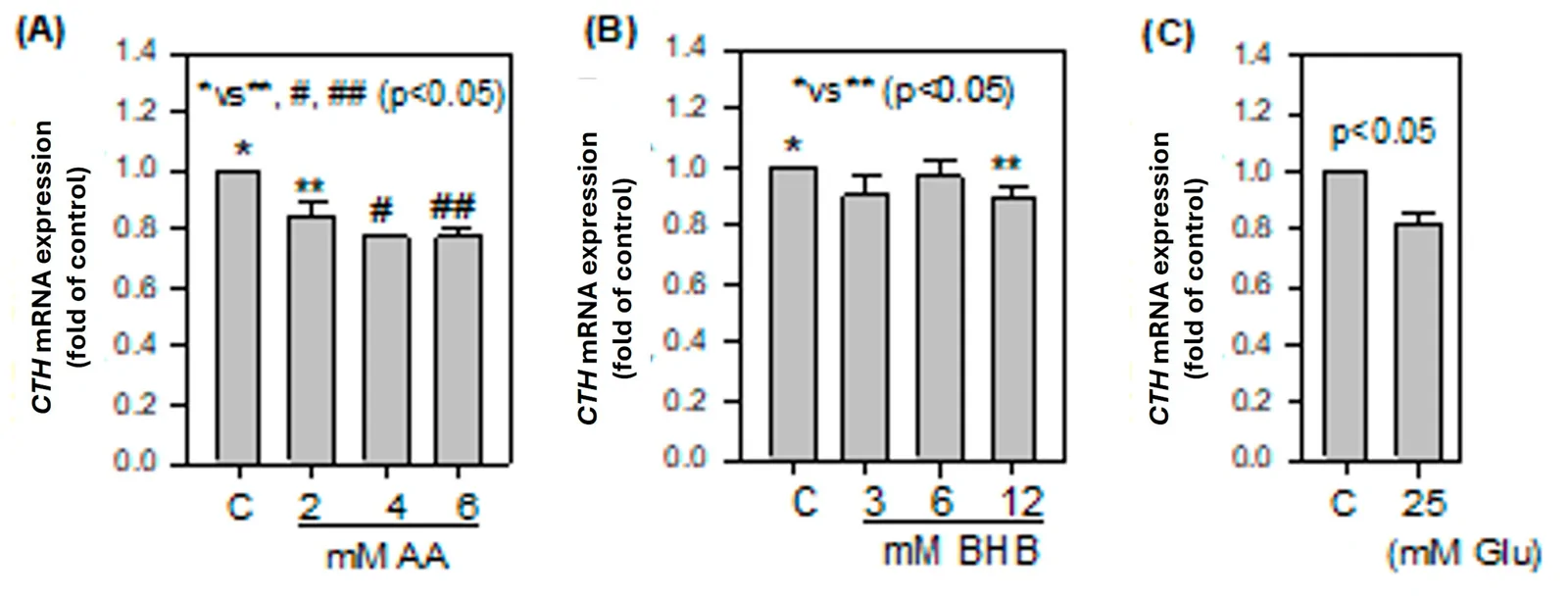
Effects of (**A**) AA, (**B**) BHB, and (**C**) HG treatment on *CTH* mRNA expression in HepG2 cells. Values are mean ± SE from three independent biological experiments.

The effects of HG, AA, and BHB on *CTH* expression are shown in Figure 3. Exposure of hepatocytes to HG alone significantly reduced *CTH* gene expression (*p* < 0.05) compared with the corresponding controls. High-glucose exposure was also associated with reduced *CTH* mRNA expression compared with normal glucose conditions. None of the treatments significantly affected cell viability.

## 4. Discussion

The present study found that circulating plasma H_2_S/sulfide concentrations were significantly lower in children and adolescents with type 1 diabetes (T1D) compared to age-matched healthy controls. Within the T1D cohort, lower plasma H_2_S/sulfide concentrations were associated with higher ketone concentrations, lower HDL-cholesterol and 25(OH)VD levels, and higher MMP-9 levels. Cellular experiments further demonstrated that exposure to high glucose and acetoacetate was linked to reduced *CTH* mRNA expression. These findings suggest a connection between altered sulfide homeostasis and metabolic and inflammatory abnormalities in T1D. However, since the clinical component of the study was observational, these associations should not be interpreted as evidence that hyperketonemia directly causes reductions in circulating H_2_S. Collectively, these findings identify impaired H_2_S biosynthesis as a potential mechanism linking hyperketonemia to decreased *CTH* gene expression and vascular inflammation in T1D. This interpretation aligns with experimental evidence indicating that disrupted H_2_S homeostasis is associated with endothelial dysfunction and vascular injury [7,8,23,25].

Patients with T1D frequently experience chronic hyperglycemia and recurrent episodes of hyperketonemia, particularly during periods of inadequate insulin availability. While ketone bodies are important alternative energy substrates in scenarios of limited glucose availability, their biological effects depend on the metabolic context and circulating concentrations [24]. Increasing evidence suggests that pathological elevations of ketone bodies can influence oxidative stress, inflammatory signaling, and vascular function [2]. Specifically, acetoacetate has been shown to stimulate reactive oxygen species (ROS)-dependent activation of NF-κB and p38 MAPK signaling in endothelial cells, leading to increased expression of the adhesion molecule ICAM-1. Our findings extend these observations by demonstrating a significant inverse relationship between circulating ketone levels and H_2_S concentrations, suggesting that hyperketonemia may be linked to impaired endogenous H_2_S biosynthesis in children with T1D.

H_2_S is increasingly recognized as an endogenous gasotransmitter involved in regulating vascular tone, redox homeostasis, cellular bioenergetics, and inflammatory signaling [26]. It is primarily produced through the actions of CSE, cystathionine β-synthase (CBS), and 3-mercaptopyruvate sulfurtransferase (3-MST), with the relative contributions of these enzymes varying among different tissues [23,26]. CSE is particularly abundant in the cardiovascular system and plays a significant role in H_2_S production within blood vessels [23]. Studies have shown that *CTH* knockdown downregulates glucose transporter type 4 (GLUT4) and the gene expression of its key transcription factors, resulting in reduced glucose uptake in C2C12 myotubes. Upregulating H_2_S levels can positively affect glucose homeostasis by activating the PGC-1α/FNDC5/irisin signaling pathway in myotubes [27]. These findings provide molecular insights into the significance of the CSE/H_2_S system in maintaining cellular glucose homeostasis in C2C12 myotubes [28]. Therefore, normalizing *CTH* is essential for hydrogen sulfide production related to glucose homeostasis. Cell culture studies indicate that supplementation with 1,25(OH)(2)-VD significantly increases the gene expression of cystathionine-γ-lyase and H_2_S formation while decreasing reactive oxygen species in high-glucose-treated U937 monocytes [29]. This suggests that VD supplementation may enhance *CTH* and H_2_S formation while reducing oxidative stress, indicating that VD deficiency could lead to impaired H_2_S signaling and a higher incidence of vascular inflammation in T1D [29].

Experimental studies suggest that H_2_S supplementation or pharmacological H_2_S donors can alleviate oxidative stress, improve endothelial function, and limit the progression of atherosclerotic lesions [30,31]. In a recent experimental study, an H_2_S donor restored endothelial nitric oxide-dependent vasorelaxation under hyperglycemic conditions, further supporting the interaction between H_2_S signaling and vascular endothelial homeostasis [31]. This study expands the existing body of evidence by demonstrating reduced circulating H_2_S concentrations in pediatric T1D and an inverse association between H_2_S and hyperketonemia, suggesting that impaired H_2_S signaling may contribute to early vascular abnormalities linked to metabolic stress in T1D.

The observed changes in circulating H_2_S should be examined within the larger context of alterations in circulating metabolic mediators related to diabetes and its complications. Recent case-control studies have shown significant differences in circulating irisin concentrations among individuals with type 2 diabetes mellitus, particularly concerning the presence or absence of non-alcoholic fatty liver disease [32]. This underscores the potential of circulating metabolic mediators to reflect metabolic changes and disease-associated phenotypes. Likewise, our findings suggest that circulating H_2_S is linked to metabolic and inflammatory parameters in children and adolescents with T1D. Although H_2_S and irisin serve distinct biological functions, and the current study was not intended to compare these mediators directly, these observations advocate for further investigation of circulating signaling molecules as potential biomarkers for metabolic and vascular abnormalities associated with diabetes.

A notable finding from the cellular experiments was the reduction in *CTH* mRNA expression following exposure to high glucose and acetoacetate. Since this study did not directly assess CSE protein levels, enzymatic activity, or cellular H_2_S, the observed reduction in *CTH* mRNA cannot be interpreted as direct evidence of reduced CSE enzymatic activity or H_2_S biosynthesis. The cellular data indicate that both high glucose and acetoacetate were linked to reduced *CTH* mRNA expression under the experimental conditions employed. Because combined high-glucose and ketone treatments were not assessed, the current findings do not clarify whether these effects are additive or synergistic. Additional experiments utilizing factorial treatment designs will be necessary to explore whether hyperglycemia and hyperketonemia interact to influence *CTH* expression. This observation is consistent with prior studies indicating that elevated glucose levels reduce endogenous H_2_S production and *CTH* expression in endothelial cells [30,33]. Furthermore, hyperglycemia increases mitochondrial ROS generation, which can boost H_2_S consumption and contribute to endothelial dysfunction [30]. In this context, it is plausible that oxidative stress induced by acetoacetate may play a role in suppressing *CTH* gene expression or activity; however, this mechanism was not directly demonstrated in the current study. Further research is needed to explore ROS generation, *CTH* transcriptional regulation, post-translational modifications, and H_2_S consumption to elucidate the molecular pathway linking hyperketonemia to impaired H_2_S biosynthesis.

Reduced circulating H_2_S concentrations were associated with elevated plasma MMP-9 levels and decreased HDL-cholesterol concentrations in hyperketonemic patients. Hydrogen sulfide inhibits high-glucose-induced MMP-9 protein synthesis by activating AMP-activated protein kinase in renal epithelial cells [34]. Moreover, previous studies have reported that H_2_S prevented endometrial overgrowth in a rat model of T1D by modulating the PPARγ/mTOR and Nrf-2/NF-κB pathways [35]. MMP-9 plays a role in extracellular matrix degradation and vascular remodeling and has been implicated in various diabetic vascular complications, including diabetic retinopathy [36,37]. Experimental studies have indicated an inverse relationship between H_2_S availability and MMP-9 in diabetes; diabetic animals show increased MMP-9 alongside diminished H_2_S production, while restoring H_2_S signaling can alleviate pathological vascular remodeling [38]. Notably, prior studies have reported that elevated levels of the ketone body acetoacetate can increase lipid peroxidation and decrease glutathione (GSH) levels in the red blood cells of individuals with type 1 diabetes. Additionally, there is a positive correlation between circulating H_2_S and HDL-cholesterol concentrations, providing clinical evidence for an association between H_2_S homeostasis and HDL metabolism. The negative correlation between H_2_S and MMP-9, along with the positive association between H_2_S and HDL-cholesterol in this study, suggests that reduced H_2_S availability may be linked to a more pro-inflammatory and vascular-risk phenotype in T1D. Although differences in selected inflammatory biomarkers, including CRP, were observed between groups, not all inflammatory markers demonstrated similar changes. These findings suggest that the relationship between altered H_2_S metabolism and inflammation in T1D may be selective or dependent on the specific inflammatory pathway examined. Therefore, the present data do not establish a generalized anti-inflammatory effect of H_2_S or a direct causal relationship between reduced H_2_S and inflammation.

Another finding of this study is the association between reduced circulating 25(OH)VD concentrations and impaired H_2_S homeostasis. Deficiency in 25(OH)VD is relatively common among children and adolescents with T1D; a 2024 systematic review and meta-analysis of 6995 participants estimated that approximately 45% had 25(OH)VD deficiency, although substantial heterogeneity existed among studies and geographic regions [39]. The treatment with exogenous H_2_S and nitrite (NO_2_) upregulated the mRNA expression of 25(OH)VD biosynthesis genes (*CYP2R1* and *CYP27B1*) in cultured monocytes [34]. This study suggests a potential link between lower H_2_S levels and 25(OH)VD deficiency in T1D.

**Figure 4:**
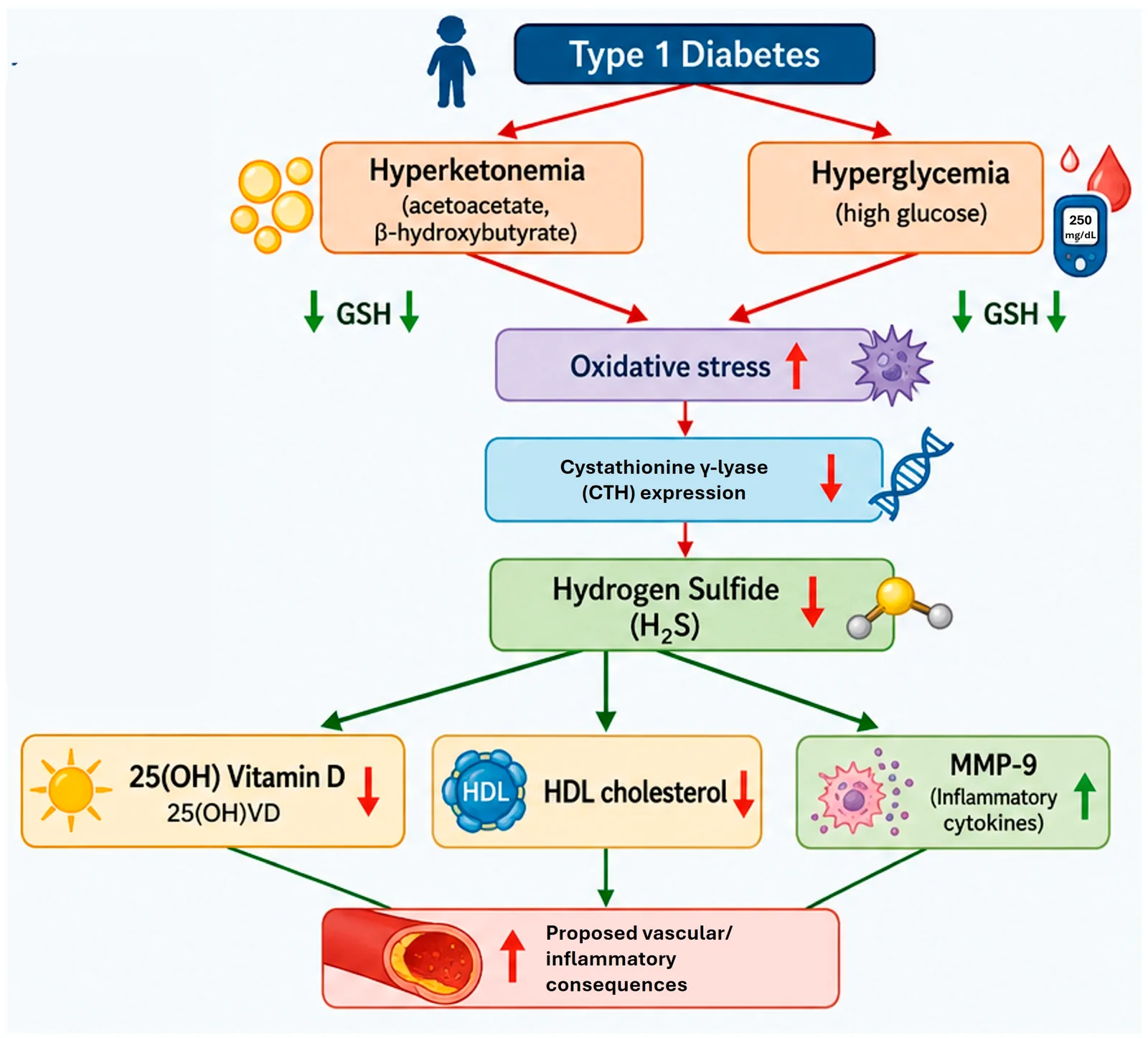
Proposed model illustrating potential associations between ketone metabolism, *CTH* expression, circulating H_2_S, and metabolic and inflammatory biomarkers in T1D. Up-arrows (↑) represent upregulation; down-arrows (↓) represent downregulation.

The status of 25(OH)VD has been linked to inflammatory and metabolic abnormalities in type 1 diabetes (T1D), although the clinical significance of these associations is not fully established [40]. In this study, we observed that circulating H_2_S concentrations were positively correlated with 25(OH)VD levels, while both biomarkers exhibited an inverse relationship with MMP-9 levels. These findings suggest a potential link between vitamin D status, H_2_S homeostasis, and inflammatory signaling. Experimental evidence supports the biological plausibility of this interaction. For instance, 1,25-dihydroxyvitamin D_3_ has been shown to enhance CSE activation and H_2_S production in 3T3-L1 adipocytes exposed to high glucose, indicating that 25(OH)VD signaling may regulate H_2_S biosynthesis through the CSE pathway [41]. Furthermore, treatment with 25(OH)VD has been found to decrease oxidative stress and inflammatory mediators in monocytes subjected to high glucose. Collectively, these observations imply that 25(OH)VD may influence inflammatory and vascular responses, at least in part, by preserving CSE/H_2_S signaling. However, since the evidence linking vitamin D, H_2_S, and MMP-9 comes from a combination of observational and experimental studies, further mechanistic and clinical investigations are needed to ascertain whether modulation of the vitamin D–CSE/H_2_S axis has therapeutic relevance in T1D. This study reveals for the first time that both hyperglycemia and hyperketonemia contribute to a reduction in *CTH* expression and activity, which may lead to impaired H_2_S signaling associated with diabetes [19] (Figure 4).

Several limitations must be considered when interpreting these findings. Firstly, the clinical component of the study was observational and, as such, cannot establish a causal relationship between hyperketonemia and circulating H_2_S concentrations. Secondly, the number of participants, particularly in the hyperketonemic subgroup, was relatively small, which limits the statistical power for subgroup and multivariable analyses and increases the potential for residual confounding. Healthy controls were recruited from families attending the pediatric diabetes clinic, which may introduce selection or familial factors, thereby limiting the generalizability of the findings. Future studies should aim to recruit controls independently from the diabetes clinic and, where feasible, from the same source population as the T1D participants. Thirdly, multiple associations were examined; therefore, appropriate corrections for multiple comparisons were applied, and any statistically significant findings should be interpreted with caution. Fourthly, the methylene blue assay provides an operational measurement of the sulfide pool captured under the specific assay conditions and should not be construed as a direct measurement of free bioactive H_2_S. Fifthly, the in vitro experiments were conducted using HepG2 cells rather than primary human hepatocytes, and only *CTH* mRNA expression was measured. CSE protein abundance, enzymatic activity, and cellular H_2_S production were not assessed directly. Lastly, the study did not evaluate clinical vascular outcomes; hence, the relationship between altered H_2_S metabolism and actual vascular disease risk remains to be determined.

## 5. Conclusions

Hydrogen sulfide is an antioxidant molecule that exhibits a wide range of biological effects against oxidative stress and has the potential to prevent endothelial cell injury [42,43] and cognitive decline [44,45]. In the present case–control study, children and adolescents with T1D exhibited lower circulating levels of plasma H_2_S/sulfide and 25(OH)VD compared to healthy controls. Within the T1D cohort, lower plasma H_2_S/sulfide levels were linked to higher ketone concentrations, reduced HDL cholesterol and 25(OH)VD levels, and elevated MMP-9 levels. In cultured hepatocytes, exposure to high glucose and acetoacetate resulted in decreased expression of *CTH* mRNA. Collectively, these findings indicate that altered ketone metabolism may be related to changes in the CSE/H_2_S pathway in individuals with T1D. However, the observational nature of the clinical study, along with the measurement of CBS only at the mRNA level in the cellular experiments, limits the ability to draw conclusions about causality or direct impacts on CSE protein, enzymatic activity, or H_2_S production. Further research is necessary to elucidate the molecular mechanisms behind these associations and to determine whether modulation of the CSE/H_2_S pathway could have therapeutic implications in T1D. Recent findings discussing polygenic enrichment of common variant signals provide evidence of a link between H_2_S biology and cerebrovascular disease [46]. These findings offer new insights into the genetic influences on H_2_S signaling and its relevance to human disease, highlighting genetic variation as a tool to prioritize indications for H_2_S-targeted therapies [46]. In addition, the potential role of L-cysteine supplementation in increasing H_2_S availability remains a hypothesis for future experimental and clinical research and was not assessed in the current study.

## Figures and Tables

**Table 1:** Primer sequences used for qPCR gene expression.

Gene	Orientation	Primer Sequence (5′-3′)
*CTH*	Forward	CCCCACAAACCCCACCCAGAA
	Reverse	GACACCAGGCCCATTAC
*GAPDH*	Forward	AGTATGACAACAGCCTCAAGAT
	Reverse	GTCCTTCCACGATACCAAA

**Table 2:** BMI and blood levels of glycemia and ketonemia in normal and T1D children. Values are mean ± SE.

Parameters	T1D	Normal
*n*	36	35
Age (yr)	13.8 ± 0.4	14.1 ± 0.5
M/F	18/18	18/17
BMI	22.6 ± 0.7	21.0 ± 0.8
Duration of Diabetes (yr)	5.6 ± 0.7	n/a
Glucose (mg/dL)	192.3 ± 12.7	78 ± 1.3 *
HbA1C (%)	9.9 ± 0.3	5.2 ± 0.04 *
Acetoacetate (mM)	0.9 ± 0.1	0.67 ± 0.09 *
β-Hydroxybutyrate (mM)	23.3 ± 0.8	14.1 ± 1.1 *
Total Ketone (mM)	24.2 ± 0.8	14.7 ± 1.1 *
Hydrogen Sulfide (µM)	17.6 ± 2.6	20.5 ± 2.0 *
25(OH)vitamin D (nM)	59.06 ± 2.5	70.8 ± 2.51 *

BMI, body mass index; n/a, not applicable; T1D, type 1 diabetes; * *p* < 0.05.

**Table 3:** Blood levels of inflammatory biomarkers in normal, T1D, normoketonemic, and hyperketonemic-T1D subjects. Data are presented as mean ± SE.

Parameter	Normal	T1D	NK-T1D	HK-T1D
*n*	35	36	25	11
Age (yrs)	14.1 ± 0.5	13.8 ± 0.4	13.8 ± 0.4	13.7 ± 0.91
CRP (ng/mL)	58.02 ± 7.8 *	73.4 ± 9.2 **	65.9 ± 9.1 ^#^	91.7 ± 22.6 ^##^
H_2_S (µM)	20.5 ± 2.0 ^	17.6 ± 2.6 ^^	20.1 ± 3.5 ^§^	11.77 ± 2.3 ^§§^
TNF-α (pg/mL)	0.31 ± 0.04 *	0.4 ± 0.04 **	0.43 ± 0.06	0.4 ± 0.04
HDL (mg/dL)	58.3 ± 2.5	54.2 ± 2.2	58.3 ± 2.5 ^#^	44.9 ± 2.75 ^##^
MMP-9 (ng/mL)	18.02 ± 0.7	17.9 ± 0.9	17.7 ± 1.1	18.3 ± 1.6
TG (mg/dL)	78.3 ± 7.2	81.6 ± 6.7	78.3 ± 7.2	89.4 ± 15.1

CRP, C-reactive protein; MMP-9, Matrix Metalloproteinase-9; TNF-α, Tumor Necrosis Factor; H_2_S, hydrogen sulfide; HDL, high-density lipoprotein; TG, triglycerides; *p* < 0.05—* vs. **; ^#^ vs. ^##^; ^ vs. ^^; ^§^ vs. ^§§^.

## Data Availability

The datasets used and/or analyzed during the current study are available from the corresponding author upon reasonable request.

## Abbreviations

The following abbreviations are used in this manuscript:

25(OH)VD	25-hydroxyvitamin D
BHB	β-hydroxybutyrate
BMI	Body mass index
CSE	Cystathionine-γ-lyase
GSH	glutathione
H2S	Hydrogen sulfide
HDL	High-density lipoprotein
HK	Hyperketonemic
MMP-9	matrix metalloproteinase-9
NK	Normoketonemic
T1D	Type 1 diabetes
